# Psychiatric disorder in prolonged post-concussive syndrom : clinical assessment, physiopathology and management review of the literature

**DOI:** 10.1192/j.eurpsy.2024.1030

**Published:** 2024-08-27

**Authors:** I. Bouguerra, A. Touiti, A. Hajri, A. Maamri, H. Zalila

**Affiliations:** Outpatient and Emergency Psychiatric Department, Razi Hospital, Manouba, Tunisia

## Abstract

**Introduction:**

According to the American Psychiatric Association’s Diagnostic and Statistical Manual of Mental Disorders, Fifth Edition (DSM-5), post concussive syndrome (PCS) is given a diagnosis of either major or mild neurocognitive disorder (NCD) due to traumatic brain injury TBI. However Persistent post-concussion symptoms (PPCS) are more complex, and typically involve multidisciplinary assessment and management. The symptoms are varied, non-specific and the therapeutic process is defiant for psychiatrist.

**Objectives:**

To investigate the semiology of persistent post-concussion syndrome (PPCS) and the therapeutic challenges it poses.

**Methods:**

A literature review was made on Pubmed, Google Scholar and Cochrane library using keywords: “post-concussive syndrome”,”psychiatric disorder”, “depression”,”post-traumatic stress disorder”,”treatment”, “physiopathology”.

**Results:**

The physiopathology of persistent PCS is controversy.The Symptoms are due to the Concept of “Symptom Generators” which results from the alterations in neurophysiology and neuropathology secondary to the injury, and pre- or post-injury psychological factors physiological concussion. The Global cerebral metabolic disturbance, the autonomic nervous system dysfunction and the cerebral blood flow dysregulation induce biochemical cascade,excitotoxic reaction and immunotoxicity.

Clinical diagnoses associated with PPCS are:Major depressive disorder,Post traumatic stress disorder,Anxiety disorder,Substance abuse disorder,Psychotic disorder and Antisocial personality disorder. For the non pharmacological management: A systematically early information and a graded physical exercise in addition to other treatment are essential.

Antidepressant, benzodiazepine and mood-stabilizer are the most recommended treatments for psychiatric symptoms. Atypical neuroleptics are indicated in delirant disorder, behavior disorder and antisocial personality disorder.Some studies suggest the methylphenidate and biperiden to treat several cognitive impairment and severe behavior disorder.

**Conclusions:**

(PPCS) is far from being a subjective complaint by patients. It is a complex clinical entity that groups symptoms that overlap with other psychiatric diagnoses, such as depression, post-traumatic stress disorder, and mood disorders. Early neuropsychiatric assessment and personalized pharmacological and psychotherapeutic treatment are essential factors in the prognosis of the disease.

**Disclosure of Interest:**

None Declared

